# Therapeutics

**Published:** 1879-01

**Authors:** 


### THERAPEUTICS.

RAPID RELIEF OF THE VIOLENCE OF ASTHMA BY THE HYPODERMIC USE OF MORPHIA.—By Dr. Henri Huchard. (*L'Union Médicale*, July 25 and 27, and October 3, 15, and 24, 1878.)

In several articles in the above mentioned journal, Dr. H. Huchard has strongly advocated the use of hypodermic injection of morphia in cases of asthma. Having found such marked relief in the spasmodic efforts at respiration, he argues that it exerts a special influence on the respiratory system. On this hypothesis, he pursued its application in other affections where the difficulty of respiration becomes a troublesome symptom, namely, in aortic affections, accompanied with cerebral anæmia, phthisis, and uræmia. After the publication of his first papers, Dr. H. received interesting cases from other physicians, in which its effects were equally happy.

Four well marked cases of relief of labored respiration, associated with interstitial nephritis, are carefully detailed.

In the concluding article, Dr. H. answers the following questions :

1. Can the hypodermic injection of morphia be substituted by the opium preparations, or by morphia introduced into the alimentary canal ? Assuredly not.

2. Does not the frequent hypodermic injection of morphia produce serious inconvenience ? Not when carefully performed. He begins with from 0.003 to 0.005 grams, increasing the dose to 0.01 or 0.015 grams at the most for each paroxysm.

3. Is it in virtue of its hypnotic action that morphia relieves the dyspnœa, or has it a direct action on the respiration ? Mr. H. claims, however opinion may differ as to its physiological action, clinical experience establishes the fact that morphine facilitates respiration ; *la morphine fait respirer.*

4. Does the morphine medication only relieve the symptom dyspnœa, without any influence on the duration of the disease which is the cause of the symptom ? This question is referred to further observation, but the pain being relieved, the general system is necessarily benefited, and the disease thus probably modified.

FOR TOOTHACHE. (*The Doctor.*) Mr. Merson uses the following formula :

| | | |
|---|---|---|
| ℞ | Chloroform. pur | 12 |
| | Tr. aconiti (Fleming) | 12 |
| | Tr. capsici | 4 |
| | Tr. pyrethri | 2 |
| | Ol. caryoph | 2 |
| | Gum. camph | 2 |
| | Misce. | |

He abolishes the pain of extraction by first drying the gum and tooth, and then applying four or five drops of the above on a little cotton. Then, having previously *warmed the forceps*, extract without delay. For toothache, a pellet of wool soaked in the above may be introduced into the cavity, and often gives instant relief.

SALICYLIC ACID AS AN ANTAPHRODISIAC. (*The Doctor*, Oct. 1, '78.)

Dr. Jewett and others have observed that salicylic acid appears to possess great power as an antaphrodisiac. Some patients who have taken it, are reported to have lost sexual power for months. If these statements prove correct, the drug may be made very useful. Gram doses of sodic salicylate five times daily are said to have abolished the power for three months.

TREATMENT OF NOCTURNAL ENURESIS.—(*The Doctor.*)

Kelp treats this affection by weak doses of nitrate of strychnia injected subcutaneously in the region of the sacrum. One or two injections, the second some days after the first, are usually sufficient.

---

ENORMOUS CONSUMPTION OF QUININE IN THE RUSSIAN ARMY. —The Russian military hospitals have consumed, during 1877, six thousand kilogrammes of the sulphate and three thousand kilogrammes of the chloro-hydrate of quinine. Enormous as this consumption is, it will be exceeded by that of the current year.—*Lyon Méd.*

